# Venesection as a Means for the Arrest of Unavoidable Hemorrhage in Placenta Prævia

**Published:** 1869-03

**Authors:** O. C. Gibbs

**Affiliations:** Frewsburg, Chautauqua Co., N. Y.


### Correspondence.

### Venesection as a Means for the Arrest of Unavoidable Hemorrhage in Placenta Prævia.

Medical literature teems with diverse and contrary teachings. That doctors differ, has long been proverbial, and it is painful, as well as humiliating, to reflect that they differ on matters of the most vital importance. Let a person, uninitiated into the mysteries of medical literature and practice, undertake to study up any important disease that afflicts humanity, and he will soon become painfully bewildered with the most positive assertion of pathological and therapeutic opinions, not only dissimilar, but directly antagonistical. With the thoroughly educated, observing and practiced physician, these contradictions are, perhaps, productive of no harm, and, possibly may be of some utility in stimulating inquiry and a constant alertness to discriminate the false from the true in theory and practice.

These thoughts were suggested on reading a paper entitled "Venesection as one of the Means for the Arrest of Unavoidable Hemorrhage," by C. C. F. Gay, M. D., of Buffalo, N. Y., and published in the *American Journal of the Medical Sciences* for January, 1869.

It has never been my great misfortune to see a case of *placenta prævia*, though in active practice for over twenty years; and, even with the light shed upon the subject by Dr. Gay fresh in mind, and his new "means for the arrest of unavoidable hemorrhage," I do most sincerely hope I never may. I have seen very alarming uterine hemorrhages, and my mind can now recall the ghastly pallor, the faintings, gaspings, and labored breathings of such, and also my alarm and anxiety. As they now present themselves to my mind's eye, I would as soon put a lancet into a corpse as to open a vein in the arm of one of these. Though I have neglected this important "means," I will say I have yet my first fatal case to record of uterine hemorrhage. If I had a cider barrel that I found leaking from a worm-hole in one head, I would as soon think of stopping it by boring a two inch auger hole in the other, as to stop hemorrhage from the uterus by opening a vein in the arm. It is true, we hear much about derivatives; but is not the two inch auger hole equally a derivative, to or from the leaking worm hole? and is not, in that case, the flow from the latter greatly diminished, but will the barrel be emptied any the slower? or would the former leak necessarily stop, even if the larger hole were subsequently plugged?

But it is not my purpose to discuss the propriety or impropriety of venesection, in the cases referred to by Dr. Gay; but to attempt to show the utter worthlessness of his premise and recklessness of reasoning.

Dr. Gay bases his advocacy of venesection in unavoidable hemorrhage, upon a single case of "*placenta prævia lateralis*," in which a vein was opened, eight ounces of blood taken, *and the patient did not die!* Now let us see his reasoning upon this one case: "I claim that it will, *beyond a doubt, at least, in a majority of cases, arrest* uterine hemorrhage." Will he please tell us *how* he knows this *without a doubt?* His next sentence is, "IF it will do this in *five* cases out of *ten*, the woman is certainly entitled to the benefit." Most "certainly;" but that "*if*" is embarrassingly in the way, notwithstanding he asserts positively that it, (venesection,) will do *more* than this. He assumes a statement to be a fact, and reasons from that assumption. Again, we ask, *how* he knows his *assumption* to be a *fact?* Still more absurd than the above, he says: "*I* have no difficulty in *believing* that the arrest of blood might be made complete by the abstraction of eight ounces of blood from the arm, provided always the patient had not lost already such an amount of blood as to have produced syncope, and I am *clearly* of the opinion that the physician would be justified in so acting, even though he did not accomplish the arrest of flooding with the lancet, *for* the chances in his favor for *success* against *failure* MIGHT be in the ration of *ten* to *one*, and this certainly should be sufficient justification!" Again, I say "*certainly*," if he will remove that *might be* by the certainty of reliable facts. I might as well say, *do not bleed* in such cases, for death *might* be the immediate results, in *ten* cases to *one* recovery, "and this should certainly be sufficient justification" for withholding the lancet.

This case, upon which Dr. Gay bases his advice of venesection as one of the means of arresting unavoidable hemorrhage, occurred, the doctor tells us, some sixteen years ago; since which he has seen, in consultation, some four or five other cases, in neither of which did he *recommend* or *practice* venesection, though some of them died. This fact alone suggests the idea that the *case* did not suggest the propriety of the treatment at the time, neither did such treatment occur to him, until after seeing the last of the other cases of placenta prævia. The idea occurred to him, evidently, within the last year, and then he conveniently remembers the case of sixteen years before. This view is sustained by the doctor's own statements, previously made. Dr. Gay reported this same one case, occurring in his practice, and treated by venesection, before the *Buffalo Medical Association*, April 7th, 1868, which elicited strictures and comments at the time, from other members. The whole proceedings were published in the *Buffalo Medical and Surgical Journal* for May, 1868. Then and there Dr. Gay said, "I am *certainly not* an advocate of venesection in placenta prævia; should *not advise* it, and probably shall never have occasion to resort to it again, and probably should not have recourse to it if I had occasion, but in the case reported, occurring in my own practice some years ago, when venesection was more fashionable than now, it were folly to say that the end did not justify the means."

Now I am prepared to assert and maintain just this "folly." The case was, by admission, one of placenta prævia lateralis, and evidently but a very small portion of the placenta overlapped the os, and was detached in the process of dilitation of the os uteri. On examination he found the "os pretty well dilated," and "pains vigorous," and yet he "decided to bleed at once!" flooding was "at once arrested," and "labor was soon completed by the efforts of nature!" Now what arrested the hemorrhage? Was it the venesection? By no means. The patient was just entering upon the second stage of labor; the vertex caught and compressed the detached portion of the placenta, and the vigorous pains completed the labor "by the efforts of nature." All the agency the doctor had in the matter, was to retard *convalescence* just so long as it took nature to restore the eight ounces of blood unnecessarily lost! The reason his imprudent treatment did not *kill* his patient was, because she had not previously lost sufficient blood as to make the loss of eight ounces more necessarily fatal! But the doctor may tell us that the hemorrhage did not come from the detached portion of the placenta, but from the *deplacented* (if I may be allowed to coin a new word) uterus. It makes no difference. The rapidly advancing vertex could compress that portion of the uterus as well. I do not propose to discuss whether the blood, in such cases, comes from the uterus or placenta. As well might we discuss whether, in case of wound in the thigh, the blood comes from the heart or the great toe! It may come from above or it may come from below the wound, as a vein or artery is severed. So in placenta prævia, if lateral more especially, it may come from the placenta, or from the uterus, or both.

It should have been observed that from the date at which the doctor says, in the Buffalo Medical Association, that he is certainly *not an advocate of venesection* in *unavoidable hemorrhage*, to the time when he *does so advocate* it in the *American Journal of the Medical Sciences*, he has had no further experience at all in placenta prævia, or with venesection as a means to arrest the hemorrhage in the same. This shows conclusively that the advocacy of the means is not deduced from the case, but the case conveniently remembered to support the advocacy. In view of this fact, my readers can judge for themselves how much importance can be placed in his statement when he says, "I have never seen any evidence that this local flowing in the least facilitates expansion, while on the other hand, are we not surrounded by a 'cloud of witnesses,' to testify to the power of venesection in overcoming a rigid os?" Now I have seen an abundance of such "evidences," and presume most other observing physicians have done the same. The doctor's two papers, taken in connection, unfortunately impress one that he first decided to advocate a measure, and then conveniently remembered or disremembered facts or circumstances to support or justify that measure. This idea finds additional confirmation in the fact that, as his case was first reported, it was criticised somewhat: as he remembers the case in his second report, he avoids some points of criticism. I will illustrate by quoting a few passages:

| *Buffalo Medical and Surgical Journal,* May, 1868. | *Amer. Journal of the Medical Sciences,* January, 1869. |
|---|---|
| "Date not recorded in my note book." | "In March, 1852." |
| "Aged 28 years." | "Aged 27 years." |
| "Had *three* children; no difficulty at their birth." | "Mother of *two* children; former labors normal and easy." |
| "She had taken the upright position.' | "*Sitting* nearly in the upright position." |
| "Os *partially* dilated." | "Os *pretty well* dilated." |
| "Pains *vigorous*, and at every pain *profuse* hemorrhage." | "Her pains were regular, but *not vigorous;* she had lost considerable blood during her pains." |
| "The *emergency* seemed *pressing*, and not knowing what better to do, resorted to venesection." | "Gave *ergot*, and *waited* until I ascertained that at each pain the blood spirted freely from the uterus." |

It will be remembered that, when the doctor made his first report, he was severely criticised for not giving ergot, and in his defence he did not then remember that he had! It takes ergot full half an hour for its full effects. Though he now remembers that he gave it, he does not tell us how long he waited; but we may infer not long, as he says, "I decided to bleed at once."

The doctor's study of placenta prævia was, doubtless, mainly made since he made his first report, and since seeing the last of his cases. The Rev. Henry Ward Beecher has given us instances in which he claims a man may conscientiously *remember what never happened*, and possibly the doctor's report, at least, may be an evidence of another case in point.

O. C. Gibbs, M. D.,
Frewsburg, Chautauqua Co., N. Y.

